# Transmission of Alzheimer's Disease-Associated Microbiota Dysbiosis and its Impact on Cognitive Function: Evidence from Mouse Models and Human Patients

**DOI:** 10.21203/rs.3.rs-2790988/v1

**Published:** 2023-04-28

**Authors:** Yiying Zhang, Yuan Shen, Ning Liufu, Ling Liu, wei li, Zhongyong Shi, Hailin Zheng, Xinchun Mei, Chih-Yu Chen, Zengliang Jiang, Shabnamsadat Abtahi, Yuanlin Dong, Feng Liang, Yujiang Shi, Leo Cheng, Guang Yang, Jing X. Kang, Jeremy Wilkinson, Zhongcong Xie

**Affiliations:** Massachusetts General Hospital; Tenth People’s Hospital of Tongji University; Massachusetts General Hospital; Massachusetts General Hospital; Massachusetts General Hospital; Massachusetts General Hospital; Massachusetts General Hospital; Geriatric Anesthesia Research Unit, Department of Anesthesia, Critical Care and Pain Medicine, Massachusetts General Hospital and Harvard Medical School; Department of Anesthesiology, Columbia University

**Keywords:** Alzheimer's disease, gut microbiota, transmission, short chain fatty acid, cognitive function, pathogenesis

## Abstract

Spouses of Alzheimer’s disease (AD) patients are at higher risk of developing AD dementia, but the reasons and underlying mechanism are unknown. One potential factor is gut microbiota dysbiosis, which has been associated with AD. However, it remains unclear whether the gut microbiota dysbiosis can be transmitted to non-AD individuals and contribute to the development of AD pathogenesis and cognitive impairment. The present study found that co-housing wild-type mice with AD transgenic mice or giving them AD transgenic mice feces caused AD-associated gut microbiota dysbiosis, Tau phosphorylation, and cognitive impairment. Gavage with Lactobacillus and Bifidobacterium restored these changes. The oral and gut microbiota of AD patient partners resembled that of AD patients but differed from healthy controls, indicating the transmission of oral and gut microbiota and its impact on cognitive function. The underlying mechanism of these findings includes that the butyric acid-mediated acetylation of GSK3β at lysine 15 regulated its phosphorylation at serine 9, consequently impacting Tau phosphorylation. These results provide insight into a potential link between gut microbiota dysbiosis and AD and underscore the need for further research in this area.

## One Sentence Summary

The study found evidence supporting the transmission of gut microbiota dysbiosis and its impact on cognitive function from Alzheimer's disease (AD) patients to non-AD (their partners/caregiver), highlighting a potential link between gut microbiota dysbiosis and AD pathogenesis.

## Introduction

Alzheimer’s disease (AD) is the most prevalent dementia among the elderly, with approximately 6.2 million cases in the United States and 24 million worldwide (1). Despite extensive research, effective treatments and preventative measures for AD have remain elusive. The pathological hallmarks of AD includes accumulation of beta-amyloid (Aβ) (2), phosphorylation of Tau proteins [(3–6), reviewed in (7, 8)], and neuroinflammation (9–11). However, the upstream modulators of these pathological features and the underlying mechanism remain unclear.

Gut microbiome (GMB) plays a significant role in brain functions and behavior via the gut-brain axis (12–14), and gut microbiota dysbiosis have been linked to AD patients and AD pathogenesis (15–18). Differences in GMB have been observed between AD patients and healthy controls (17). Characteristic alterations in GMB in AD patients include reduced levels of Eubacterium rectale, Firmicutes and Bifidobacterium, and increased levels of Escherichia/Shigella, Bacteroidetes and Proteobacteria compared to healthy control (19–21). Animal studies have also shown differences in GMB in AD mouse models, including 5XFAD (15, 22), APPSwe/PSEN1dE9 (23–25), and APPSwe/PSEN1L166P (26) strains, compared to WT mice. Brandscheid et al. reported increased Firmicutes and decreased Bacteroidetes phyla in 5XFAD mice at 9 weeks compared to WT mice (27). However, Chen et al. reported the opposite pattern of decreased Firmicutes and increased in Bacteroidetes phyla at 3 months in 5XFAD mice compared to WT mice (15).

Clinical studies have demonstrated that spouses of AD patients have a higher risk of developing incidental AD dementia (28–33). Furthermore, stress and emotional well-being can also have a significant impact on the GMB (34). The GMB and progression of AD can be regulated by diet (1, 35–38), sleep (39–41) and exercise (42, 43). These findings suggest that environments factors may contribute to the incidental AD dementia observed in spouses of AD patients. However, no studies have compared GMB among AD patients, spouses of AD patients and healthy controls. Notably, clinical studies can be challenging due to the required long follow-up time and confounding factors, making it crucial to understand these potential differences of GMB among AD Tg mice, WT mice co-housed with AD Tg mice and WT mice without co-housing with AD Tg mice.

Recent studies have shown that the GMB may play a role in regulating β-amyloid (Aβ) accumulation and neuroinflammation in AD and other neurological conditions (44). Specifically, antibiotics treatment has been shown to alter the GMB composition and Aβ accumulation in AD mouse models (45). Fecal matter transplants (FMT) from non-antibiotics treated AD transgenic (Tg) mice to antibiotics-treated AD Tg mice restores the GMB and Aβ accumulation, suggesting a link between GMB changes and Aβ accumulation (46, 47). Furthermore, antibiotics or germ-free (GF), which can mediate GMB depletion, have been shown to alter microglial inflammatory state (45, 46, 48–50). Antibiotics also reduce plaque-associated microglia and change microglial morphology (45, 46, 50). However, the transmission of AD pathogenesis and cognitive impairment from AD to non-AD mice through co-housing, as well as the underlying mechanisms, remains unknown.

Considering that GMB impact AD-associated pathogenesis, it is logical to hypothesize that WT mice co-housed with AD Tg mice obtain AD-associated pathogenesis and develop cognitive impairment through acquiring AD-associated gut microbiota dysbiosis. The present study defined this hypothesis by showing that WT mice co-housed with AD Tg mice, referred to as AD-exposed WT (ADWT) mice, acquired AD-related gut microbiota dysbiosis and developed AD pathogenesis and cognitive impairment. The study also revealed that the mechanism underlying this effect that butyric acid, a short-chain fatty acid produced by GMB, mediated acetylation-regulated phosphorylation in GSK3β, a kinase known to be involved in the phosphorylation of Tau (51, 52). Additionally, our clinically relevant studies indicated that the partners of AD patients (PAD) also acquired GMB profiles similar to AD patients, but distinct from

non-AD controls (CON).

## Results

### WT mice developed cognitive impairment after co-housing with AD Tg mice.

**Figure 1:**
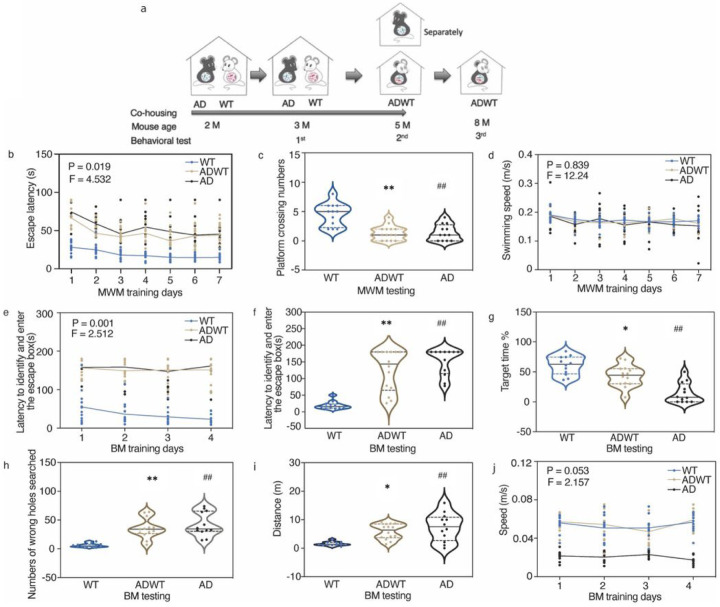
WT mice developed cognitive impairment after co-housing with AD Tg mice. **a.** The 2-months-old WT mice co-housed with 2-months-old AD Tg mice for up to 3 months are defined as ADWT mice. The ADWT mice were separated from AD Tg mice at age of 5-months-old. The behavioral tests of mice were performed at age of 3, 5 and 8 months old. After co-housing with AD Tg mice for 3 months, the AD Tg mice and ADWT mice developed cognitive impairment compared to the WT mice, as demonstrated in increased MWM escape latency during training days **(b)**, decreased MWM platform crossing numbers on testing day **(c)**, but no significant changes in swimming speed in MWM **(d)**, increased latency to enter escape box during BM training days **(e)**, and increased latency to enter escape box on BM testing day **(f)**, decreased BM target time on testing day **(g)**, increased number of wrong holes searched on BM testing day **(h)**, increased distance on testing day **(i)**, but no significant changes in speed during training days **(j)** compared to the WT mice. Data are mean ± standard deviation or medians (with interquartile ranges), N = 12–14 mice in each experimental group. The P values refer to the differences of variables among the groups, * P < 0.05; ## P < 0.01. Two-way ANOVA with repeated measurement and Bonferroni correction was used to analyze the data presented in b, d, e, and j. The P values refer to the interaction of group in MWM and BM training days. One-way ANOVA with Bonferroni correction was used to analyze the data presented in c, f, g, h, and i. AD, Alzheimer’s disease; WT, wild-type; ADWT, AD-exposed WT; Tg, transgenic; MWM, Morris water maze; BM, Barnes maze.

**Figure 2:**
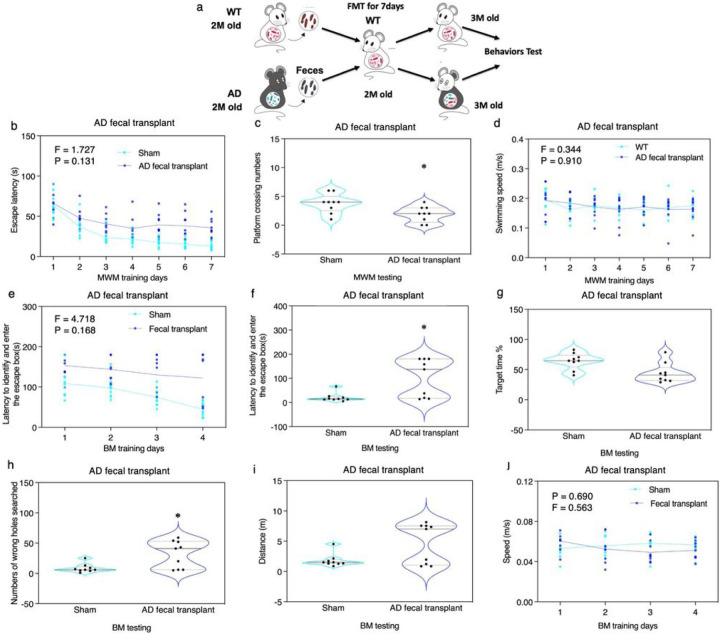
WT mice developed cognitive impairment after fecal microbiota transplantation from AD Tg mice. **a.** The 2-months-old WT mice received gavage of fecal microbiota from the 2-months-old WT or AD Tg mice for 7 days; the behaviors of the recipient WT mice were tested one month after the gavage at 3-months-old. The recipient WT mice received AD mice fecal microbiota transplantation developed cognitive impairment compared to the WT mice received saline, as demonstrated as increased escape latency during training days **(b)**, decreased platform crossing number on testing day **(c)**, but no significant changes in swimming speed **(d)** of MWM. The recipient WT mice received AD mice fecal microbiota transplantation developed cognitive impairment compared to the WT mice received saline, as demonstrated in increased latency to enter escape box during BM training days **(e)**, increased latency to enter escape box on BM testing day **(f)**, increased number of wrong holes searched on BM testing day **(h)**, but not significant changes in BM target time on testing day **(g)**, no significant changes on distance on BM testing day (i), and no significant changes on speed during training days. N = 9 mice in each experimental group. Two-way ANOVA with repeated measurement and Bonferroni correction was used to analyze the data presented in b, d, e, and j. The P values refer to the interaction of group in MWM and BM training days. One-way ANOVA with Bonferroni correction was used to analyze the data presented in c, f, g, h, and i. The P values refer to the differences of variables between the groups, * P < 0.05. AD, Alzheimer’s disease; WT, wild-type; Tg, transgenic; MWM, Morris water maze; BM, Barnes maze; FMT, fecal microbiota transplantation.

**Figure 3:**
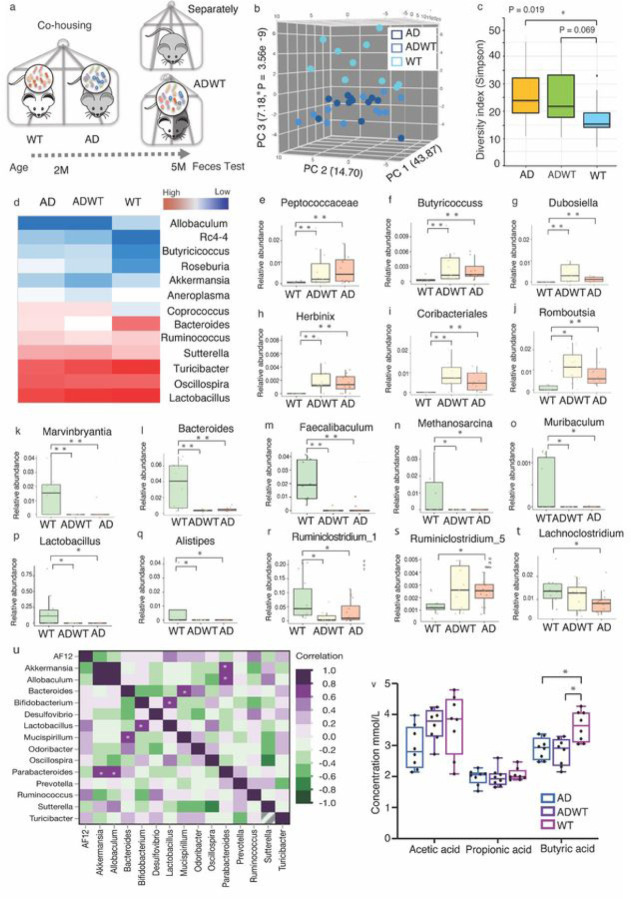
ADWT mice acquired AD-associated gut microbiota dysbiosis after co-housing with AD Tg mice. **a.** Experimental design of fecal collection after 3 months co-housing. **b.** Principal component analysis (PCA) using the Bray–Curtis dissimilarity metric among fecal samples of WT, ADWT, and AD Tg mice (P = 3.56e-9; permutational multivariate analysis of variance, PERMANOVA). Each dot represents an individual. PC1, PC2, and PC3 represent the percentage of variance explained by each coordinate. **c.** The Simpson diversity of ADWT mice was similar to that of AD Tg mice, but different from that of WT mice. **d.** Heatmap indicated the different changes in bacterial community structure represented as relative abundance shown in genus level analysis among the AD Tg, ADWT and WT mice. Relative to the WT mice, the AD Tg and ADWT mice had higher relative abundance of Peptococcaceae**(e)**; Butyricoccuss **(f)**; Dubosiella **(g)**; Herbinix **(h)**; Coribacteriales **(i)**; Romboutsia **(j)**, but lower relative abundance of Marvinbryantia **(l)**; Bacteroides **(m)**; Faecalibaculum **(n)**; Methanosarcina **(o)**; Muribaculum (p); Lactobacillus **(q)**; Alistipes **(r)**; Ruminclostridium_1 **(s)**. Relative to the WT mice, the AD Tg mice had higher relative abundance of Ruminclostridium_5 **(k)** but lower relative abundance of Lachnoclostridium **(t)**. **u**. The heat map demonstrated the pair-wise correlative relationship between any pair two bacteria among the WT, ADWT, and AD Tg mice. h. Changes in fecal short chain fat acids showed that the AD Tg and ADWT mice had less butyric acid compared to the WT mice had. N = 8–12 biologically independent samples in each group. * P < 0.05; ** P < 0.01. MaAsLin 2 implementation were used for testing in microbiome profiles taxonomic result (e to t). AD, Alzheimer’s disease; WT, wild-type; PC, principal component.

We co-housed two-month-old female WT mice with the same age and gender AD Tg mice for a period up to 3 months, referred to hereafter as the AD-exposed WT (ADWT) mice (Fig. 1a). After the 3-month of co-housing with AD Tg mice, both the ADWT mice and AD Tg mice developed cognitive impairment compared to WT mice. Specifically, the ADWT and AD Tg mice had longer escape latency during the training days (Fig. 1b) and fewer number of platforms crossing on the testing day (Fig. 1c) in Morris water maze (MWM) than the WT mice. There was no significant difference in swimming speed of MWM among these three groups of mice (Fig. 1d). Similarly, in Barnes maze (BM) test, relative to the WT mice, the ADWT and AD Tg mice had longer time to identify and enter the escape box during the training days and on the testing day of BM (Fig. 1e, 1f), reduced target zone entrances (Fig. 1g), more wrong holes searched before entering on the escape box on the testing day of BM (Fig. 1h), and longer distance of BM on the testing day (Fig. 1i). There was no significant difference in speed between the ADWT and WT mice in BM, but the AD mice showed the trend of decreased speed compared to the WT and ADWT mice (Fig. 1j). The cognitive impairment in ADWT mice was sustained for at least 3 months after the co-housing ended (**S-Fig. 1**). Notably, the WT mice co-housed with AD Tg mice for 1 month did not lead to cognitive impairment assessed in MWM and BM (**S-Fig. 2**), and the AD Tg mice co-housed with WT mice (WTAD), did not show the improved cognitive function compared to the AD Tg mice (**S-Fig. 3**). These results suggest that the WT mice co-housed with AD Tg mice, the ADWT mice, can develop a time-dependent (1 versus 3 months) and long-term (up to 3 months) cognitive impairment.

**Figure 4:**
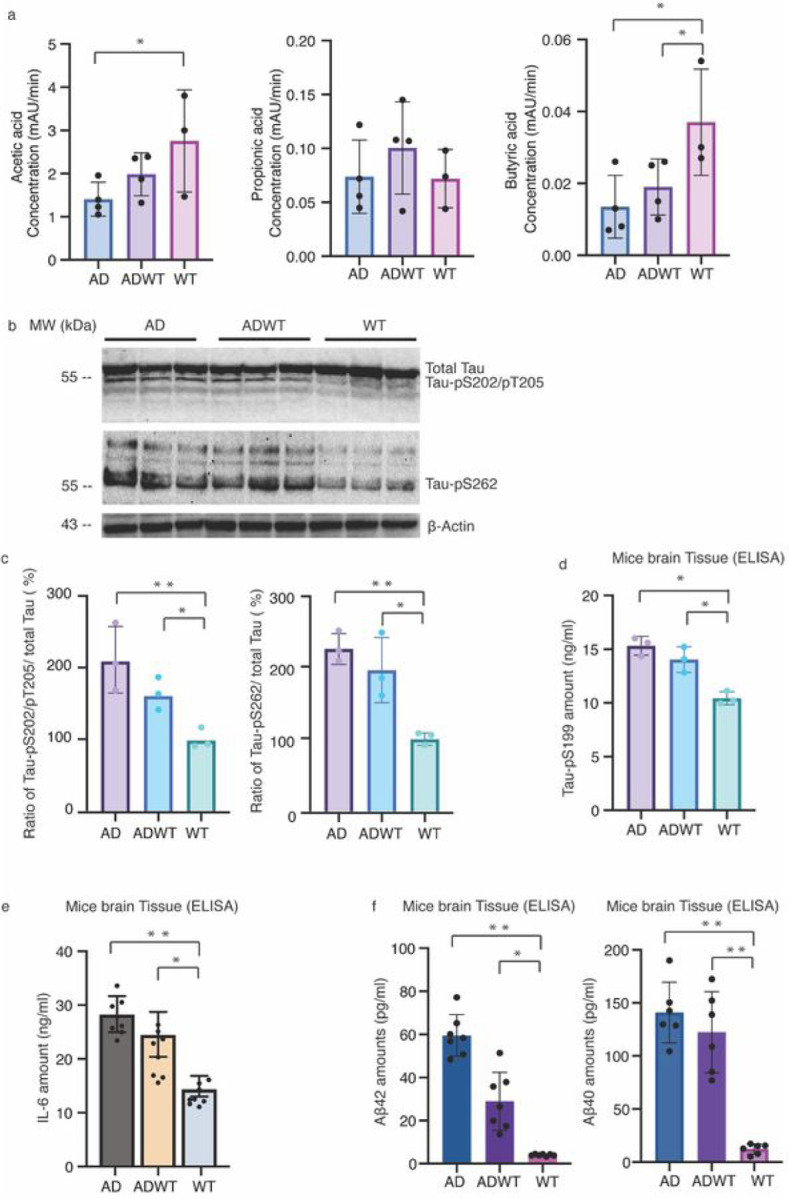
Differences in brain levels of SCFAs, phosphorylated Tau, IL-6 and Ab among AD Tg, ADWT and WT mice. *a.* The AD Tg mice had less brain acetic acid, but not propionic acid, levels compared to WT mice. Both the AD Tg and ADWT mice had less butyric acid levels in brain tissues compared to the WT mice. *b*. Western blot shows that the AD Tg and ADWT mice had higher amounts of Tau-pS202/PT205 and Tau-pS262, but not total Tau, in the hippocampus compared to WT mice. *c.* The quantification of the Western blots showed that the AD Tg and ADWT mice had a higher ratio of Tau-pS202/PT205 to total Tau and Tau-pS262 to total Tau in the hippocampus compared to the WT mice. *d*. ELISA showed that AD Tg and ADWT mice had higher Tau-pS199 amounts in the hippocampus compared to WT mice. *e.* The AD Tg and ADWT mice had higher amounts of IL-6 in the hippocampus compared to WT mice. *f*. The AD Tg and ADWT mice had higher amounts of Ab42 and Ab40 in the hippocampus compared to the WT mice. N = 3 – 8 biologically independent samples in each group. One-way ANOVA with *Bonferroni* correction was used to analyze the data presented in a, c, d, e, f and g. * *P* < 0.05; ** *P* < 0.01. AD, Alzheimer’s disease; WT, wild-type; Tg, transgenic; Interleukin 6, IL-6.

**Figure 5:**
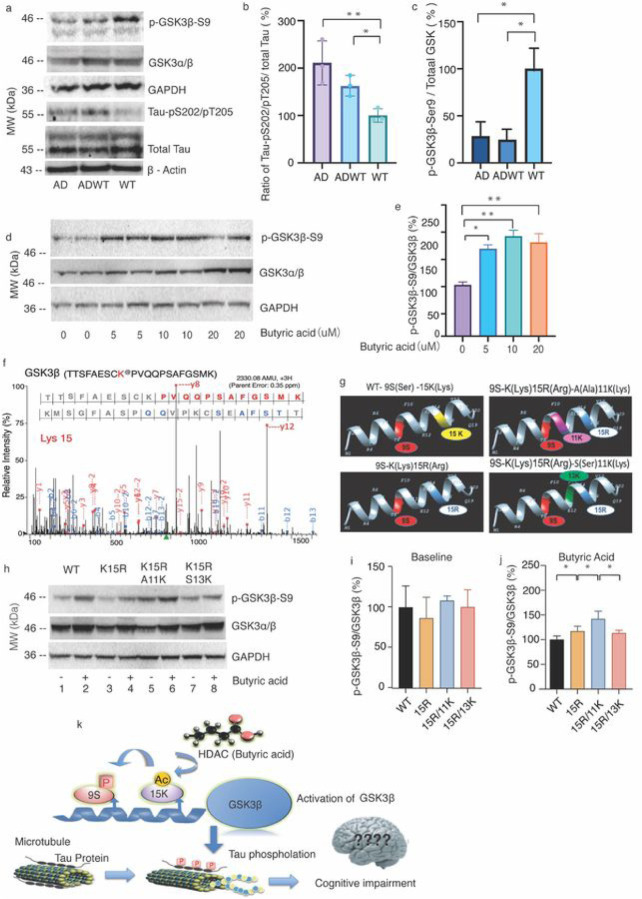
Butyric acid increased GSK3b-S9 levels dependent on acetylation of lysine at 15. **a.** Western blot showed that the AD Tg and ADWT mice had higher amounts of Tau-pS202/pT205 and lower amounts of GSK3b-S9 in the hippocampus compared to WT mice. ***b.*** The quantification of the Western blot demonstrated that AD Tg and ADWT mice had higher ratio of Tau-PS202/PT205 to total Tau in the hippocampus compared to WT mice. ***c*.** The quantification of the Western blot demonstrated AD Tg and ADWT mice had lower ratio of p-GSK3b-S9 to total GSK3b in the hippocampus compared to WT mice. ***d*.** Western blot showed that the butyric acid induced a dose-dependent increase in p-GSK-3b-S9 levels in HEK293T cells and LY2090314 (LY), the inhibitor of GSK-3, blocked the effect of butyric acid. ***e*.** The quantification of the Western blot demonstrated the dose-dependent effects of butyric acid on increasing the ratios of p-GSK-3b-Ser9 to GSK3bin HEK293T cells, LY block the effect of butyric acid. ***f.*** Annotation of representative tandem mass spectra of Trypsin-GluC digested GSK3b, depicting K15 acetylation following the treatment of butyric acid. ***g.*** The computer-generated WT and 3 independent site-directed mutations (K15R, K15R/A11K, and K15R/S13K). *h*. The effects of butyric acid on amounts of p-GSK3b-Ser9 and GSK3b in WT and the 3 independent site-directed mutants (K15R, K15R/A11K, and K15R/S13K) HEK293T cells. ***i*.** The 3 mutations did not significantly change the base line ratios of p-GSK-3b-Ser9 to GSK3b. ***j*.** However, the mutations of K15R increased the ratios of p-GSK-3b-Ser9 to GSK3b; the mutations of K15R plus A11K had greater, but the mutations of K15R plus A13S had lesser, increases in the ratios of p-GSK-3b-Ser9 to GSK3bthan the mutations of K15R following the butyric acid treatment. ***j*.** The hypothesized pathway showing that lysine at 15 of GSK3b may play an important role in the butyric acid-mediated inhibition of GSK3bactivity, Tau phosphorylation and cognitive impairment. N = 3 biologically independent samples in each group. AD, Alzheimer’s disease; WT, wild-type; p, phosphorylated; PS, phosphorylated serine; PT, phosphorylated threonine; LY2090314, LY; K, lysine; R, arginine; S, serine; A, alanine.

**Figure 6:**
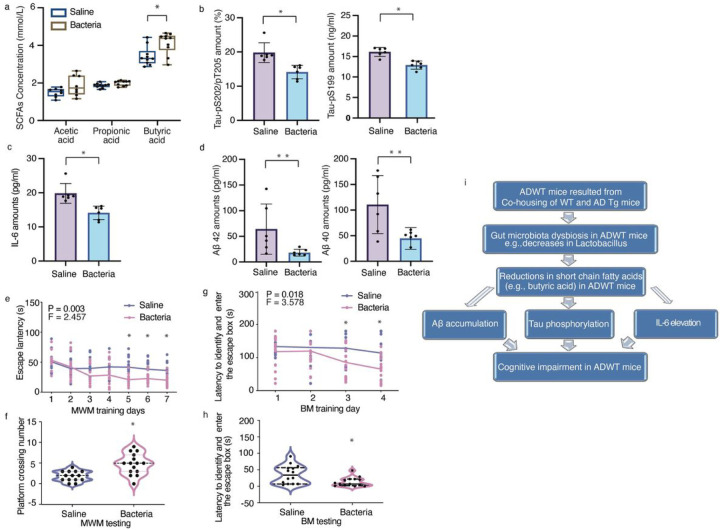
Treatment with bacteria (*Lactobacillus* and *Bifidobacterium*) mitigated the behavioral and cellular changes in ADWT mice. ***a.*** The gavage of *Lactobacillus*plus *Bifidobacterium* (in the first 10 days in each month of the total 3 months) increased fecal butyric acid amounts of the ADWT mice compared to saline treatment. The gavage of *Lactobacillus* plus *Bifidobacterium* reduced the amounts of Tau-pS202/pT205 and Tau-pS199 **(*b*)**, IL-6 **(*c*)** and Ab42 and Ab40 **(*d*)** in the hippocampus of the ADWT mice compared to saline treatment. Finally, the ADWT mice with gavage of *Lactobacillus* plus *Bifidobacterium* had better cognitive function compared to the ADWT mice with saline treatment, as demonstrated in MWM training **(*e*)**, MWM testing **(*f*)**, BM training **(*g*)**, and BM testing **(*h*)**. ***i***, The hypothesized pathway showing that ADWT mice, resulting from the co-housing of AD Tg and WT mice, acquire the AD-associated gut microbiota dysbiosis, which causes reductions in gut and brain butyric acid amounts, leading toTau phosphorylation, IL-6 elevation and Ab accumulation, leading to the cognitive impairment in the ADWT mice. Data are mean ± standard deviation or median (with interquartile range), N = 6 to 15 biologically independent samples in each group. Student’s t-tests were used to analyze the data in a, b, c, and d. Two-way ANOVA with repeated measurement and *Bonferroni* correction was used to analyze the data presented in e and g. Mann Whitney U test was used to analyze the data in f and h. AD, Alzheimer’s disease; WT, wild-type; p, phosphorylated; pS, phosphorylated serine; pT, phosphorylated threonine; Interleukin 6, IL-6; MWM, Morris water maze; BM, Barnes maze.

The study also conducted fecal microbiota transplantation (FMT) experiments to validate the observed cognitive impairment was due to coprophagia, the re-ingestion of feces, by the ADWT mice. Two-month-old female WT mice were administered with FMT, obtained from two-month-old female AD Tg or WT mice, for seven days (Fig. 2a). Results showed that the WT mice that received fecal microbiota from AD Tg mice developed cognitive impairment evidence in MWM (Fig. 2b–d) and BM (Fig. 2e–j), while those that received microbiota from WT mice did not (**S-Fig. 4**). Further experiments ruled out the confounding influence of airborne transmission and environment on the observed behavior. Specifically, we compared the behavior of mice which had air exchange or in different location. Neither indirect contact via air exchange between AD Tg and WT mice (**S-Fig. 5**) nor housing of WT mice in a different location for 3 months (**S-Fig. 6**) caused cognitive impairment in the WT mice. These data suggest that active (co-housing) or passive intake (FMT) of AD Tg mice feces can induce cognitive impairment in the WT mice.

### WT mice acquired gut microbiota dysbiosis after co-housing with AD Tg mice.

**Figure 7:**
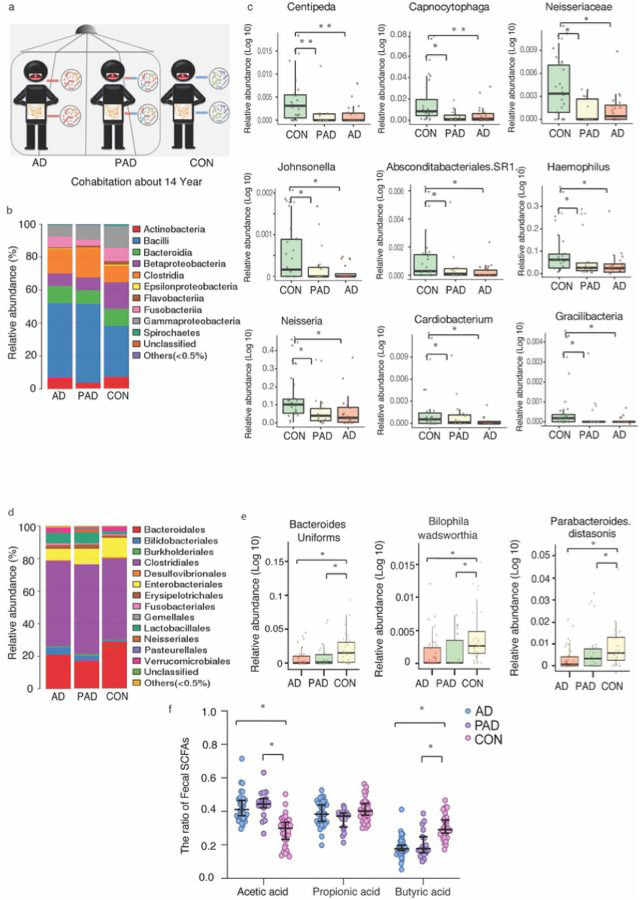
Microbiota in oral and fecal samples of Alzheimer’s disease (AD) patients, partners of AD patients (PAD), and control (CON) individuals. ***a*.** Schema of oral and fecal sample collection from AD, PAD, and CON. ***b*.** The average taxonomic distribution of bacteria from oral 16S RNA sequencing at the order level among the AD, PAD, and CON. ***c.*** Relative abundances of *9 bacteria* in oral samples from AD and PAD were significantly lower than those in CON. ***d*.** The average taxonomic distribution of bacteria from fecal 16S RNA sequencing at the order level among the AD, PAD, and CON. ***e.*** Relative abundances of *3 bacteria* in fecal samples from AD and PAD were significantly lower than those in CON. ***f*.** The ratios of acetic acid and butyric acid to total SCFAs in fecal samples among the three cohorts showed that the AD and PAD had higher acetic acid, but lower butyric acid compared to CON. There were the following biologically independent samples in each group of oral samples: AD (N = 19), PAD (N = 11), CON (N = 24) and fecal samples: AD (N = 39), PAD (N = 22), CON (N = 33). * or # *P* < 0.05; ** *P* < 0.01. MaAsLin 2 implementation were used for testing in microbiome profiles taxonomic result (***c*** and ***e***). One-way ANOVA with *Bonferroni* correction (f). AD, Alzheimer’s disease; PAD, partners of AD; and CON, control.

Considering the findings that ADWT mice developed cognitive impairment potentially due to the transfer of GMB from co-housing with AD Tg mice, we then compared the GMB composition among AD Tg mice, ADWT mice, and WT mice (Fig. 3a). Principal component analysis (Fig. 3b, S-**Fig. 7a and 7b)** demonstrated that the GMB profiling of the ADWT mice (represented by sky blue dots) was similar to that of AD Tg mice (represented by dark blue dots) but different from WT mice (represented by light blue dots). Additionally, the Simpson diversity index (α-diversity) at the operational taxonomic unit (OTU) level showed that the Simpson diversity index of AD Tg mice was statistically significant and that of ADWT mice was borderline significant, both higher than that of WT mice (Fig. 3c). There were no significant differences in body weight among the three groups of mice (**S-Fig. 7c**), but the AD Tg and ADWT mice had higher levels of fecal moisture content and weight compared to the WT mice (**S-Fig. 7d, 7e)**. The heatmap in Fig. 3d showed the GMB community profile among the three groups of mice at Genus level. We then used the Microbiome Multivariable Association with Linear (MaAsLin2) Models to determine the multivariable associations among WT, ADWT, AD Tg mice and their GMB meta-omics features at species levels (53). Compared to WT mice, the GMB in the AD and ADWT mice was characterized by an increased abundance of proinflammatory bacteria *Dubosiella* (54) and six other bacteria (Fig. 3e to 3j), but decreased abundances of other bacteria (Fig. 3k to 3r), including the *Marvinbryantia* (Fig. 3k), associated with bowel dysfunction (55); anti-inflammatory bacteria *Bacteroides* (Fig. 3l) and *Lactobacillus* (56) (Fig. 3p). The bacteria associated with short-chain fatty acids (SCFAs) production (57), Faecalibaculum (Fig. 3m) and Ruminiclostridium-1(Fig. 3r), were also decreased in AD and ADWT mice compared to WT mice.

Notably, AD Tg mice (statistical significance) and ADWT mice (trending) also exhibited increased abundance of Ruminiclostridium-5 (Fig. 3s), associated with mucosa-related microbiome and obesity (58), and decreased abundance of *Lachnoclostridium* (Fig. 3t), a novel marker for colorectal cancer (59), compared to WT mice. The quantification of the bacterial taxa association for comparison between WT mice and ADWT mice or AD Tg mice at species levels was presented in **S-Table 1**. We also demonstrated the correlative relationship of top 15 bacteria at the genus level and found that *Bifidobacterium* and *Lactobacillus* were highly associated in combined data from AD Tg, ADWT and WT mice (Fig. 3u).

Finally, we observed that AD Tg mice and ADWT mice had decreased amount of butyric acid, one of SCFAs, in their feces compared to WT mice (Fig. 3v). This was consistent with the previous findings that AD Tg mice and ADWT mice had reduced abundance of *Faecalibaculum* (Fig. 3m) and *Ruminiclostridium-1* (Fig. 3r), the bacteria which generated SCFAs, compared to WT mice.

### ADWT mice exhibited reduced amounts of butyric acid, increased Tau phosphorylation, elevated IL-6 and accumulated Aβ42 and Aβ40 amounts in brain tissues.

Building on the previous findings that ADWT mice showed cognitive impairment that may have been transmitted from AD Tg mice through GMB. We further measured the levels of SCFAs in the brain tissues of mice. Our results showed that both AD Tg and ADWT mice exhibited decreases in butyric acid levels in the brain, which was in line with the decrease in feces, compared to WT mice (Fig. 4a). Additionally, we observed changes consistent with AD pathogenesis, including increased levels of Tau phosphorylation, indicated by elevated amounts of Tau-PS202/PT205, Tau-PS262, and Tau-PS199, in the hippocampus of the AD Tg and ADWT mice (Fig. 4b–4d). The AD Tg and ADWT mice also showed elevated levels of IL-6 (Fig. 4e) and accumulation of Aβ42 and Aβ40 (Fig. 4f) in the hippocampus compared to the WT mice. These findings suggest that the transmission of GMB from AD Tg mice to ADWT mice may play a role in the development of AD pathogenesis and cognitive impairment in the ADWT mice.

### Butyric acid mediated-acetylation of GSK3β regulates its phosphorylation.

Our study found that AD and ADWT mice had higher amounts of Tau-PS202/PT205 in the brain tissues compared to WT mice (Fig. 5a, 5b), which is associated with AD pathogenesis (60, 61). Our study also found that the ratio of phosphorylated (p) GSK3β-serine9 to GSK3β was lower in AD and ADWT mice (Fig. 5a, 5c) compared to WT mice. In vitro experiments showed that butyric acid increased the ratio of p-GSK3β-serine9 to GSK3β in HEK 293T cells (Fig. 5d, 5e). The results of mass spectrometry (MS) studies indicated that the acetylation of GSK3β at lysine 15 (K15) (Fig. 5f). And K15 is a critical acetylation site of in regulating the phosphorylation of GSK3β at serine 9 (Fig. 5f). Our study also found that the distance between serine 9 and the next lysine (11 versus 13) plays a critical role in regulating phosphorylation of GSK3β at serine 9. The mutation of lysine (K) 15 to arginine (R) increased GSK3β phosphorylation at serine 9 and converting Alanine (A) 11 to lysine (K) 11 further increased GSK3β phosphorylation at serine 9. On the other hand, inserting serine (S) 13 to lysine (K)13 had less effect on GSK3β phosphorylation at serine 9 than K15R/A11K following butyric acid treatment (Fig. 5g,5h, and 5i). This information could contribute to a better understanding of the role of butyric acid in regulating AD pathogenesis, including that lysine (K) 15 of GSK3β is a critical acetylation site in regulating phosphorylation of GSK3β at serine 9 following treatment of butyric acid, which then leads to alterations in Tau phosphorylation.

### Treatment with Lactobacillus plus Bifidobacterium attenuated the behavioral and cellular changes in the ADWT mice.

Given that ADWT mice had gut microbiota dysbiosis, e.g., decreased abundance of *Lactobacillus* compared to that of WT mice (Fig. 3q) and *Lactobacillus and Bifidobacterium* were highly associated in the mice (Fig. 3u), next we asked whether the treatment with *Lactobacillus* plus Bifidobacterium could attenuate the changes in the ADWT mice. We found that treatment with *Lactobacillus* and *Bifidobacterium* was associated with higher amounts of butyric acid (Fig. 6a), as well as lower levels of Tau-PS202/PT205 and Tau-PS199 (Fig. 6b); less IL-6 levels (Fig. 6c); and less Aβ40 and Aβ42 amounts (Fig. 6d) in brain tissues compared to treatment with saline in ADWT mice. Additionally, the ADWT mice treated with *Lactobacillus* and *Bifidobacterium* showed improved cognitive function to those treated with saline (Fig. 6e–6h, **and S-Fig. 9**). These data suggest that treatment with Lactobacillus and Bifidobacterium may have therapeutic benefits for ADWT mice and that the gut microbiota dysbiosis observed in ADWT mice contributes, at least partially, to the observed changes in AD pathogenesis and cognitive impairment in the mice. (Fig. 6i).

### Partners of AD patients developed AD-associated gut microbiota dysbiosis.

Finally, we determined the clinical relevance of these preclinical findings. We compared the oral and fecal microbiota among AD patients, partners of AD (PAD) living in the same household, and non-AD control, CON (community-dwelling elder) (Fig. 7a **and S-Fig. 10**). The clinical covariates were presented in detail in **S-Tables 2, S-Tables 3, S-Fig. 11, S-Fig. 12**.

The oral microbiome analysis showed the average taxonomic distribution in AD and PAD were similar in microbial compositions with higher abundances of *Bacilli* and *Clostridia*, but lower abundances in *Gammaproteobacteria* and *Betaproteobactia* compared to CON (Fig. 7b) at the orders levels. The MaAsLin2 model demonstrated the top nine bacteria the abundances of which were lower in AD and PAD than that of CON (Fig. 7c) at the species levels.

Additionally, the fecal microbiome analysis revealed that the average taxonomic distribution in AD and PAD had higher levels of *Bacteroidales* and *Lactobacillaes*, but lower levels of *Enterobacteriales* (Fig. 7d) compared to CON, at the orders levels. The fecal microbiota community in the AD and PAD was characterized by the decreases in the abundance of *Bacteroides uniforms*, which supports fiber and lipid metabolic and immune system (62, 63), compared to CON. The opportunistic pathobiont *Bilophila wadsworthia* (64) and *Parabacteroides distasonis* (65) were also found to be less abundant in AD and PAD groups compared to CON (Fig. 7e). Furthermore, the ratio of butyric acid to total SCFAs was lower, while the ratio of acetic acid to total SCFAs was higher in AD and PAD compared to CON (Fig. 7f). These data suggest that AD patients may transmit their GMB to PAD, leading to the microbiota dysbiosis in PAD. However, despite this similarity in GMB between AD and PAD, the PAD did not show significant differences in Mini-mental state exam (MMSE) scores and clinical dementia rating (CDR) compared to the CON (**S-Tables 2 and S-Tables 3**).

## Discussion

We discovered that WT mice developed gut microbiota dysbiosis because of co-habitation with AD Tg mice, refereed as ADWT mice. This microbiota dysbiosis resulted in the development of AD-associated pathogenesis and cognitive impairment in ADWT mice. Our clinical findings also indicated that partners of AD patients experienced gut microbiota dysbiosis which was similar to that of AD patients but different from that of CON. Although further investigation is required to validate these findings, our data suggests the potential transmission of GMB and related AD pathogenesis and cognitive impairment from AD to non-AD individuals. The underlying mechanism behind this GMB transmission-associated cognitive impairment may involve butyric acid, a short-chain fatty acid produced by GMB, which impacts acetylation-regulated phosphorylation in GSK3β. These changes in GSK3β may contribute to the phosphorylation of Tau protein and the subsequent cognitive impairment associated with the GMB transmission.

Clinical studies have reported that spouses of AD patients have a higher risk of developing incidental AD dementia (28–33). Consistently, we demonstrated that the WT mice co-housed with AD Tg mice developed long-time cognitive impairment (Fig. 1). Notably, the developed cognitive impairment was not due to the location or air exchange (**S-Figs. 5 and 6**). The accelerated time course in developing cognitive impairment in the ADWT mice by receiving feces from AD Tg was likely due to larger doses of bacteria introduced to the ADWT mice through the gavage feeding than they would obtain by feces-eating during the co-housing (Fig. 2). Notably, the control condition of AD FMT was saline in our study, because treatment with saline is more clinically relevant and we found that there was no significant difference between saline and heat-killed bacteria in cognitive function in the mice (data not shown). Together, these data further suggest the observed cognitive impairment from AD to non-AD may result from the transmission of GMB.

Our mechanistic studies showed that the ADWT mice acquired gut microbiota dysbiosis and developed AD-associated pathogenesis, including increased Tau phosphorylation, IL-6 amounts, and Aβ accumulation (Fig. 4). The GMB-generated metabolites can promote metabolic benefits via the gut-brain axis (66). Previous studies report that SCFAs were decreased in the feces and brain of APP/PS1 mice compared with WT mice (24). SCFAs can modulate Aβ plaques in the brain (49), astrocytic gene expression (67), expression of tight junction proteins (68), directly act on afferent vagal fibers (69), and induce ApoE-associated Tauopathy (70). In our study, butyric acid, one of the SCFAs, decreased in AD Tg and the ADWT mice compared to WT mice both in feces and brain tissues of mice (Fig. 3v and Fig. 4a). Meanwhile, the ratio of butyric acid also decreased in AD and PAD compared to CON in human studies (Fig. 7f).

Butyric acid is known to have several beneficial effects on the central nervous system, including anti-inflammatory (71) and neuroprotective effects (72). Our studies showed that butyric acid may play a role in the acetylation-regulated phosphorylation of GSK3β, leading to Tau phosphorylation. Acetylation and phosphorylation are two types of post-translational modifications that can impact the activity of enzymes, including kinases like GSK3β. Butyric acid has been shown to enhance acetylation and reduce phosphorylation of GSK3β, which can alter its activity and impact cellular processes (52). Previous studies have reported that if GSK3β are acetylated at K183, it could decrease activation of GSK3β to phosphorylate its substrates (52). Our studies identified that lysine (K) 15 is also a critical acetylation site for regulating phosphorylation of GSK3β at serine 9 and the distance between serine 9 and the next lysine (K) plays a critical role in regulating the phosphorylation of GSK3β at serine 9. As the inhibitor of histone deacetylase (HDAC), butyric acid could modify the acetylation site at K15 and decrease activation of GSK3β to phosphorylate its substrates including Tau (as shown in Fig. 5K). The data suggest K15 of GSK3β is a potential modification site for AD pathogenesis and target for interventions. Replenishment GMB with *Lactobacillus* and *Bifidobacterium* prevented the reduction of butyric acid in the ADWT mice (Fig. 6a). These findings further suggest that gut microbiota dysbiosis could reduce the amount of butyric acid, leading to Tau phosphorylation and cognitive impairment (Fig. 6i).

Germ-free mice were not used in this study because their lack of microorganisms can affect various physiological processes, including peripheral and central immune development (67), neurotransmission (73), and neurogenesis (74), which could potentially confound the study's results. Instead, AD Tg mice were co-housed with WT mice, which more closely mimics the social interactions and microorganism exchange between AD patients and their spouses that occur in a natural setting, thus leading to more clinically relevant findings.

We only used female mice in the present study because male AD Tg or WT mice tend to fight during co-housing, which would have confounded the results. We deliberately used male AD Tg mice and female WT mice to generate the AD Tg mice for the present study, avoiding any potential maternal transmission of AD-associated GMB from an AD mother to the next generation, as reported in previous studies (75–78), as confounding influence. Additionally, we did not use littermates of the AD Tg mice because they would have experienced similar co-housing conditions similar to the ADWT mice in this study, which could have introduced confounding variables into the results.

We did not find significant evidence of mucosal damage, changes in tight junction composition or structure in the small intestine or colon of AD Tg mice (**S-Fig. 8**). Therefore, it is unlikely that loss of gut barrier contributes to the changes observed in the ADWT mice in our study (79, 80). Instead, we propose that the alterations in butyric acid, which can move through the gut barrier freely, are responsible for the observed changes. To test this hypothesis further, future studies should include additional experiments.

Previous studies have reported the possibility of microbiota being transmissible among family members and social network (81),(82), and the caregivers or partners of individuals with AD may experience changes in their microbiota due to the stress associated with caregiving. These changes may contribute to symptoms of depression and other associated health problems (83). Chen et al. found that co-housing young AD Tg mice with aged AD Tg mice led to the acquisition of a similar GMB profile as that of the aged AD Tg mice, resulting in earlier onset of cognitive impairment in the young AD Tg mice (15). Valles-Colomer et al. demonstrated person-to-person transmission of the gut and oral microbiomes (84). In contrast to these studies, our work specifically confirms that GMB can be transmitted not only between AD Tg mice, but also from AD Tg mice to non-AD mice. Our results demonstrated that WT mice co-housed with AD Tg mice acquired AD-associated gut microbiota dysbiosis, leading to the development of AD pathogenesis and cognitive impairment (Figs. 1, 2 and 3). This co-housing model more closely approximates the clinical condition where partners (e.g., spouses) of AD patients co-habit with AD patients. Importantly, our study confirms that feces exchange via co-housing, rather than airborne transmission or environmental conditions, causes cognitive impairment (**S-Figs. 5 and 6**). Finally, our work also has clinical relevance, as we demonstrated that AD patients could transmit oral and gut microbiota to their non-AD partners (Fig. 7). Although we cannot exclude the contribution of diet, lifestyle, and environmental factors to GMB changes in human studies, the findings from our animal work suggest that microbiota transmission contributes, at least partially, to the observed AD pathogenesis and cognitive impairment. Our results also highlight the importance of investigating microbiota transmission in both animals and humans, particularly for the studies of non-infectious and GMB-associated diseases.

The present study has some limitations that should be acknowledged. Firstly, we did not observe any cognitive improvement in the AD Tg mice co-housed with WT mice (**S-Fig. 3**), even though the WT mice co-housed with AD Tg mice developed cognitive impairment (Fig. 1). The reason for this discrepancy is not clear, but it is possible that the 5XFAD mice used in our study had already developed an aggressive form of AD pathology, and therefore, their cognitive impairment could not be restored by the transmission of GMB from WT mice. Future studies using less aggressive AD Tg mouse models, such as the APP Tg2576 model (85) may help to further explore whether co-housing with WT mice can improve cognitive function in AD Tg mice. Secondly, although we observed a partial transfer of oral and fecal microbiota from AD patients to PAD, the PAD did not develop cognitive impairment. One possible reason for this discrepancy could be that the AD and PAD individuals did not co-habit long enough for the PAD individuals to develop incidental AD dementia. Our animal studies support this idea, as short-term co-housing (1 month) between AD Tg mice and WT mice did not result in cognitive impairment in the WT mice.

In conclusion, the present study provides evidence supporting the potential transmission of GMB from AD patients or AD Tg mice to co-housed non-AD controls, and the acquisition of AD-associated gut microbiota dysbiosis and metabolite changes as possible contributors to AD pathogenesis and cognitive impairment. Future investigations are necessary to further determine the transmissibility of AD-associated gut microbiota dysbiosis, its role in AD pathogenesis and cognitive decline, and whether targeting the GMB could be a viable intervention strategy for AD in both preclinical and clinical settings.

## Materials And Methods

Detailed methods are provided in the online version of this paper and include the following:

Key resources table

Resource availability

Lead Contact.

Materials Availability.

Data and Code Availability.

EXPERIMENTAL MODEL AND SUBJECT DETAILS

Mice model.

Human participants.

METHOD DETAILS

Mice Co-housing condition.

Microbiota transplantation.

Animal behaviors tests.

Gavage with Lactobacillus and Bifidobacterium in mice.

Harvest of mice brain tissues and collection of mice feces.

DNA extraction and quantification of relative abundance of fecal or oral bacteria.

SCFAs detection.

Enzyme-linked immunosorbent assays (ELISA).

Western blot analysis.

Cell culture and butyric acid treatment.

Mass spectrometry studies.

Plasmid constructs and transfection.

Immune staining.

Morphometry.

Human study design.

Human study participants.

Study size estimation.

## Supplementary Material

Supplement 1
