# Supplementary material for: Transmission of Alzheimer's Disease-Associated Microbiota Dysbiosis and its Impact on Cognitive Function: Evidence from Mouse Models and Human Patients
Source: Res Sq. 2023 Apr 28:rs.3.rs-2790988. Preprint. [Version 1] doi: 10.21203/rs.3.rs-2790988/v1 (PMC10168447; doi:10.21203/rs.3.rs-2790988/v1)
Supplement: Supplement 1 [file NIHPPrs2790988v1-supplement-1.pdf]

# Supplementary Files

This is a list of supplementary files associated with this preprint. Click to download.

- Supplemental.Data.Zhang.Gut.Microbiota.AD.03.13.2023.docx
